# Epidemiologic characteristics of 10 years hospitalized patients with glaucoma at shanghai eye and ear, nose, and throat hospital

**DOI:** 10.1097/MD.0000000000004254

**Published:** 2016-07-22

**Authors:** Yingying Zheng, Yuqiu Zhang, Xinghuai Sun

**Affiliations:** a Department of Ophthalmology and Visual Science, Eye and ENT Hospital, Shanghai Medical College; b Key Laboratory of Myopia, Ministry of Health and Shanghai Key Laboratory of Visual Impairment and Restoration; c State Key Laboratory of Medical Neurobiology, Institutes of Brain Science and Collaborative Innovation Center for Brain Science, Fudan University, Shanghai, People's Republic of China.

**Keywords:** glaucoma, hospitalization, proportion

## Abstract

To analyze the epidemiologic characteristics of hospitalized patients with glaucoma at Shanghai Eye and Ear, Nose, and Throat Hospital in the relative well economic condition area Eastern China.

Researchers reviewed the 10 years charts of patients with a discharge diagnosis of glaucoma at this hospital from January 2004 to December 2013. With the criteria used for diagnoses of different types of glaucoma, the constitution of this disease between the first and last 5 years was compared and analyzed.

A total of 11,864 cases were enrolled according to the criteria of diagnoses for 10 years. Primary angle-closure glaucoma (PACG) was the main type (51.69%) followed by secondary glaucoma (SG, 28.14%), primary open-angle glaucoma (POAG) (10.41%), and congenital glaucoma (7.16%). Other glaucoma types were less prevalent (2.61%). PACG has seen a declining trend (from 57.75% to 48.41%), while the proportion of SG has increased (from 23.68% to 34.21%). For the POAG group, there is no increasing trend and the same is true of CG. The mean age of PACG patients was 62.28 year-old, while it was 46 year-old for the POAG and SG groups. The patients of PACG had more women than men (M/F ratio: 1:1.92). The reverse was the case with regard to POAG (M/F ratio: 1.97:1).

In Eastern China, although PACG has some decreased, but still is the most commonly encountered type of glaucoma, while SG has significantly increased, and POAG has slightly decreased in hospitalized patients during recent 10 years.

## 1 Introduction

Glaucoma is a kind of irreversible blinding eye disease. Its pathological basis is the loss of retinal ganglion cells and the retinal nerve fiber layer which leads to optic nerve damage and visual field defects.^[1]^ The prevalence of glaucoma is increasing worldwide and will reach 79.6 million in 2020. It is impacting all countries, although China and India will be the hardest hit. Together, they will account for nearly 40% of glaucoma patients worldwide.^[2]^

The prevalence of primary glaucoma varies according to ethnicity and geographic location. In Asia and especially in China, primary angle-closure glaucoma (PACG) was regarded as the main type of primary glaucoma, but a population-based epidemiological survey shows that the prevalence of primary open-angle glaucoma (POAG) has increased. Epidemiological investigations of the residents of Beijing's Shunyi District in 1985 and 1996^[3]^ showed that the internal composition of primary glaucoma in China is changing.

In the central part of China, the majority of inpatients with glaucoma had PACG.^[4]^ This may relate to regional, economic, and cultural conditions. As for Eastern China, the relative well economic and cultural condition area, there were no reports or literature on relevant studies of hospitalized patients with glaucoma. The Eye and Ear, Nose, and Throat (EENT) Hospital of Fudan University in Shanghai was established in 1952 and is one of the biggest ophthalmological centers in China. In order to better understand the epidemiologic characteristics of glaucoma in Eastern China, the data were collected and analyzed at Shanghai EENT hospital from 2004 to 2013.

## 2 Patients and methods

A retrospective analysis was conducted of 20,779 patients hospitalized with glaucoma at Shanghai EENT Hospital from January 1, 2004 to December 31, 2013. If a patient was admitted to the hospital more than once, data would be collected only if the patient was listed as an “inpatient” at the 1st admission to the hospital. We divided our cohort into 5 groups: PACG, POAG, secondary glaucoma (SG), congenital glaucoma (CG), and other types including normal tension glaucoma, pigmentary glaucoma, exfoliative glaucoma, malignant glaucoma, and mixed glaucoma. The process of the present study and data collection conformed to the guidelines of the Declaration of Helsinki. Ethical approval was obtained from the Institutional Research and Ethics Board of the Shanghai EENT Hospital.

The criteria used for diagnoses of different types of glaucoma are as follows:

1. PACG is defined as the existence of glaucomatous optic nerve changes (loss of the optic nerve rim and defect of retinal nerve fiber layer) with glaucomatous damage of the visual field, associated with angle closured and increased intraocular pressure (IOP) and/or peripheral anterior synechia.^[5]^
2. POAG is defined as a condition in a subset of patients with open angles, and raised IOP associated with either glaucomatous damage of the optic nerve or the visual field.^[5]^
3. SG is a group of glaucoma caused by some eye diseases or systemic diseases that affect or destroy the normal aqueous humor circulation leading to elevated IOP, and optic nerve damage. Its etiology is clear, such as uveitic, neovascularization, trauma, and so on.^[5]^
4. CG can be divided to 2 types: primary CG) and secondary CG. Primary CG is a disease characterized by an isolated dysplasia of trabecular meshwork not associated with other developmental eye abnormalities or ocular diseases that can increase IOP. However, secondary CG is related to other ocular or systemic pathology that can increase IOP.^[6]^
5. Normal tension glaucoma refers to an open-angle, characteristic glaucomatous damage of the optic nerve or visual field and the IOP < 22 mm Hg without treatment.^[7]^

It should be noted that we did not include vision acuity or the history of drug treatment in this study. Only cases with a clear and confirmed diagnosis were included in the current study. All cases were examined by glaucoma doctor team members and verified by a senior glaucoma consultant.

## 3 Statistical analysis

All the data were import into a Microsoft Excel form, collating sort by 2 authors; a statistical analysis was performed in Excel. The constitution of the disease between the first and last 5 years was compared by the Pearson Chi-square test. *P* < 0.05 was considered statistically significant.

## 4 Results

**Figure 1:**
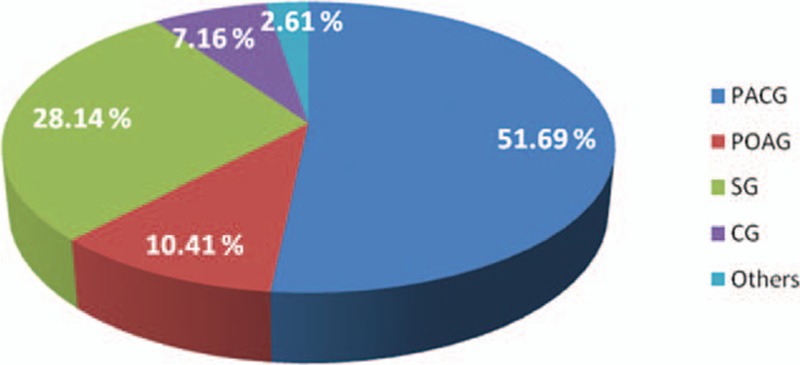
Distribution of different glaucoma types.

A total of 11,864 cases from 20,779 in-patients fulfilled the glaucoma inclusion criteria. Based on the above-mentioned criteria, the distribution of different glaucoma types is elucidated in Fig. 1. PACG comprises the largest group (6132/11,864; 51.69%) of referrals (Table 1). PACG is the most common subtype with the POAG-to-PACG ratio being 4.96:1.

**Table 1:**
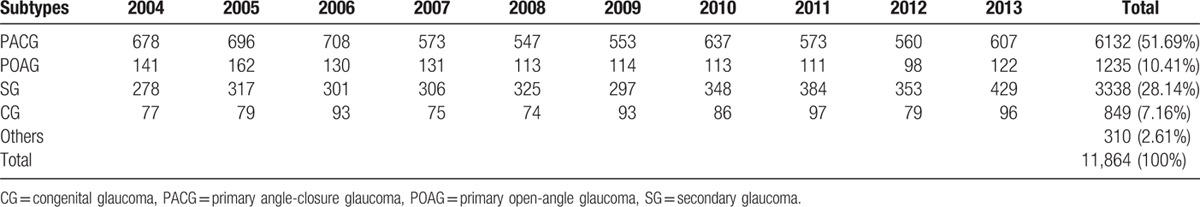
Distribution of glaucoma type.

The details of surgical age distribution in PACG and POAG are shown in Tables 2 and 3, respectively. The peak age of PACG patients was from 55 to 70 years (51.15%), while the mean age was 62.28 years (males 62.34, females 62.27). Females outnumbered males [4031/6132 (65.74%)].

**Table 2:**
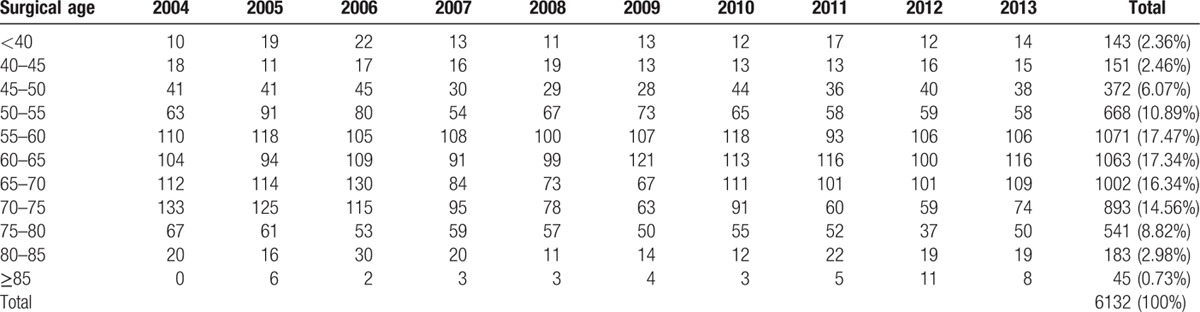
Surgical ages distribution of primary angle-closure glaucoma.

**Table 3:**
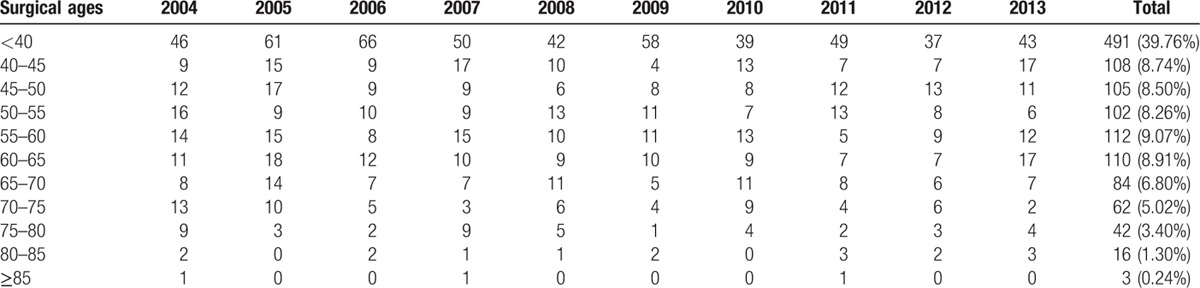
Surgical ages distribution of primary open-angle glaucoma.

SG accounted for 23.68% of patients hospitalized with glaucoma in 2004, while the percentage had up to 34.21% in 2013. The male–female ratio was 1.75:1; comparing 2004 and 2013, the sex-ratio was no significant difference after statistical processing **(**Tables 1 and 5**)**.

Inpatients with PACG and POAG were divided into 2 groups by age (<40 and ≥40). In the PACG group, females over 40 accounted for 65.85% of patients. Yet females over 40 accounted for only 35.35% of the POAG group. The results of this study showed that patients over 40 years of age accounted for 97.75% of the PACG group, but a relative lower proportion of the POAG group, 60.24% **(**Table 4**)**. The male–female ratio in each subgroup was consistent with the overall ratio.

**Table 4:**
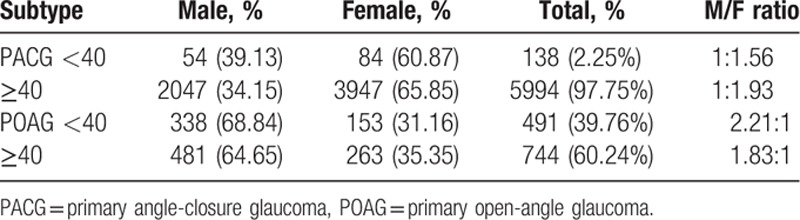
Surgical ages and gender distribution of PACG and POAG.

The details of gender distribution in different subtypes of glaucoma are shown in Table 5. The mean age of POAG patients was 45.93 (males 45.43, females 46.90). Males accounted for 819/1234 (66.32%) and females for 416/1235 (33.68%) of POAG cases **(**Table 5**)**.

**Table 5:**
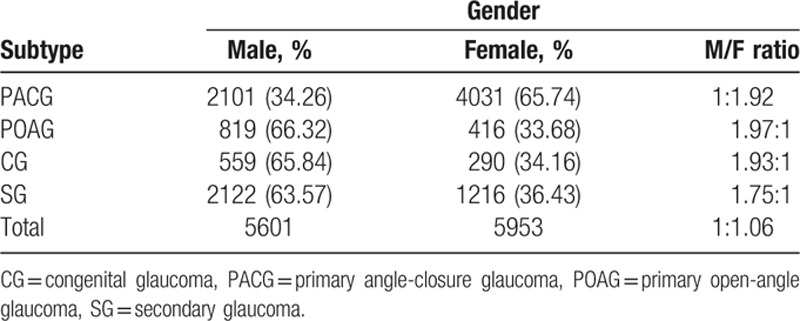
Gender distribution of glaucoma types.

The median age at diagnosis of PACG, POAG, and SG during a 10-year period is shown in Table 6. Patients with PACG were relatively older (about 62 years) than patients with other glaucoma types. However, there has been no obvious change in the median age in the past decade for all subtypes. Patients with CG between 0 and 3 years old were the most common, accounting for 43.82% **(**Table 7**)**.

**Table 6:**
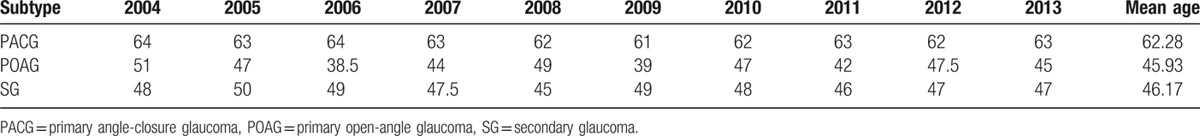
Median distribution of glaucoma types.

**Table 7:**
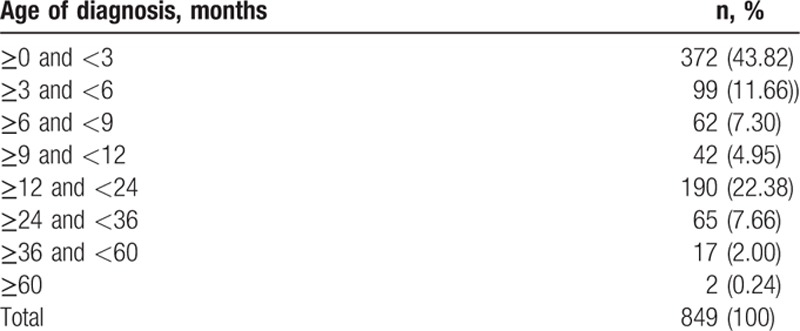
Age of diagnosis for congenital glaucoma.

**Figure 2:**
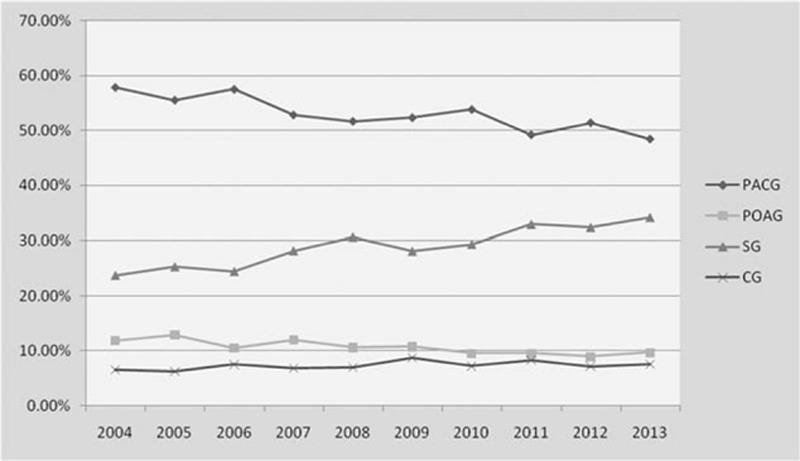
Trends in ten years of glaucoma subtypes.

For the POAG group, there is no increasing trend and the same is true of CG. PACG has seen a declining trend, while the proportion of SG has increased (Fig. 2**)**. The proportion of glaucoma changed between the first and last 5 years, and the difference was statistically significant (χ^2^ = 50.7519, *P* < 0.05). According to the results, the change may be related to PACG and SG **(**Table 8**)**.

**Table 8:**
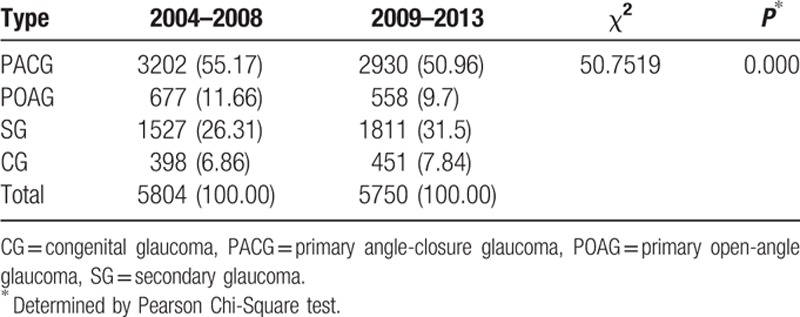
Constituent ratio between 2004–2008 and 2009–2013.

## 5 Discussion

### 5.1 The constituent ratio of glaucoma

In this study, we found PACG to be the predominant form of glaucoma in this hospital-based study in Shanghai, followed by SG. SG contributed 28.14% to all types of glaucoma in this study, while the proportion of POAG remained low. With the increasing use of prostaglandins drugs and laser therapy such as selective laser trabeculoplasty, the proportion of POAG appeared to be dropped. The constitution of CG was well preserved; comparing 2004 and 2013, the analysis showed no significant differences. CG is a kind of hereditary disease, the incidence of which is variable in different populations, and occurs in approximately 1 in every 10,000 live births.^[8]^ Many Western-based studies report POAG being far more common than PACG worldwide.^[2]^ PACG is regarded as a fairly rare disease in Western populations. The prevalence of PACG is higher in Asian populations (0.12%–2.5%) than in white populations (rare–0.6%) or among those of African descent (0.5%).^[9]^ It has been estimated that PACG may actually be a more blinding disease than POAG, at least in the Chinese population.^[10,11]^ This study shows while the proportion of PACG had declined overall the 10 years, it is still the main type of glaucoma in China. The drop of PACG was due in the increases of prophylactic YAG laser iridectomy and cataract surgeries.

### 5.2 Relationship between glaucoma and age

A previous study has shown that an estimated 3% of people over age 40 have glaucoma^[2]^ most of who are undiagnosed.^[12–16]^ This study showed the mean age of patients with PACG and POAG was 62.28 and 45.93, respectively.

Domestic research data previously showed that hospitalized primary glaucoma patients above age 40 accounted for 71.78% of all glaucoma patients, while 95.31% of PACG patients were over 40 years old.^[4]^ Our results were consistent with previous studies. Of course, there is a close relationship between PACG and age. Old age, shallow anterior chamber, narrow angle, and thickened lens forward increased the risk of PACG.^[17]^ The median age of patients with SG was younger than that of patients with PACG, but similar to POAG. There is no doubt that CG patients were the youngest, for it was diagnosed earlier than other types. Although POAG is usually concealed onset, the accurate rate of diagnosis for POAG has improved with the quick development of the diagnosis and the improvements in healthcare. The study showed main type of SG was traumatic glaucoma and neovascular glaucoma. Among them, traumatic glaucoma was closely related with male violence behaviors.

### 5.3 Relationship between glaucoma and gender

There were almost twice as many females with PACG as males, with a 1.92:1 ratio, which is between the 2.38:1 and 1.53:1 ratios reported by the Zhongshan Ophthalmic Center^[18]^ and Hebei Xingtai Eye Hospital.^[4]^ In patients with POAG, males were nearly twice as many as females, with a ratio of 1.97:1, which is consistent with previous reports. The reasons for gender difference were not clear. A number of large-scale epidemiological studies indicate that gender is not a risk factor for POAG. Previously study showed sex hormone was not associated with glaucoma.^[19,20]^ With respect to SG, including traumatic glaucoma and neovascular glaucoma, males were more prevalent than females (M/F 1.75:1). SG falls into 2 major categories: one is traumatic glaucoma related to male violence, the other is neovascular glaucoma which has a close relationship with chronic metabolic disorders, such as obesity and type 2 diabetes. Regarding the ratio of boys to girls with CG, previous studies indicated that girls are mainly affected in Japan, with a 2:3 boy-to-girl ratio. At the same time boys were more generally affected in Europe and United States; the sex ratio was 3:2.^[21–23]^ The boy-to-girl ratio in our study was 1.93:1, which is consistent with the ratio of 2.5:1 reported by Beijing Tongren Hospital. This finding suggests that the overall ratio of boys to girls in China is higher than that in the United States and Europe and contrasts with the results in Japan. In China, there were more male newborns than female newborns. Another factor is the attitude of viewing sons as better than daughters in a developing country.

Although this study cannot estimate the prevalence or the relative proportions of glaucoma in the general Chinese population, our findings may give a clue regarding fairly unique patterns of glaucoma in Eastern China region that would need to be studied further through general population glaucoma surveys. There may have been some limitations in this study, such as a potential selection bias due to the referral nature of our patients. However, we believe it is an important implication that the proportion of hospitalized patients with glaucoma is changing, and that this predominant blindness disease was PACG followed by SG, POAG, and CG in the relative well economic condition area Eastern China.
